# Iliac Apophysitis in a High School Football Player: A Case Report

**DOI:** 10.7759/cureus.110613

**Published:** 2026-06-10

**Authors:** Rock P Vomer, Emma York, Neil P Shah, George G. A Pujalte

**Affiliations:** 1 Family Medicine, Avance Care, Raleigh, USA; 2 Family Medicine, Mayo Clinic, Jacksonville, USA; 3 Research, Mayo Clinic, Jacksonville, USA; 4 Family Medicine, Avance Care, Wilmington, USA; 5 Family and Community Medicine, Mayo Clinic, Jacksonville, USA; 6 Orthopedics and Sports Medicine, Mayo Clinic, Jacksonville, USA

**Keywords:** apophysitis, conservative care, football, hip, non-contact injury

## Abstract

Iliac crest avulsion injuries are rare and frequently misdiagnosed in adolescents. We report a case of a 16-year-old boy who presented with iliac apophysitis following a noncontact injury during a football game. The diagnosis was confirmed with radiography. The patient was treated conservatively with activity modification, limited weight bearing, and physical therapy, resulting in full recovery. This case highlights the importance of considering rare apophyseal injuries and obtaining appropriate imaging in adolescent athletes.

## Introduction

Avulsion fractures of the hip and pelvis are rare injuries that most commonly affect the apophysis of the pelvis [1,2]. Patients between the ages of 8 and 14 years are most likely to experience this type of injury while participating in sports requiring many sudden direction changes [3,4]. In adolescents, the apophysis is not yet fused and therefore is susceptible to damage from cumulative stress [5-7]. Avulsions typically present after a low-impact injury without direct trauma [8]. Diagnosing this condition requires a complete physical examination and radiography [9]. Treatment is mostly conservative with occasional surgical intervention [10].

## Case presentation

**Figure 1:**
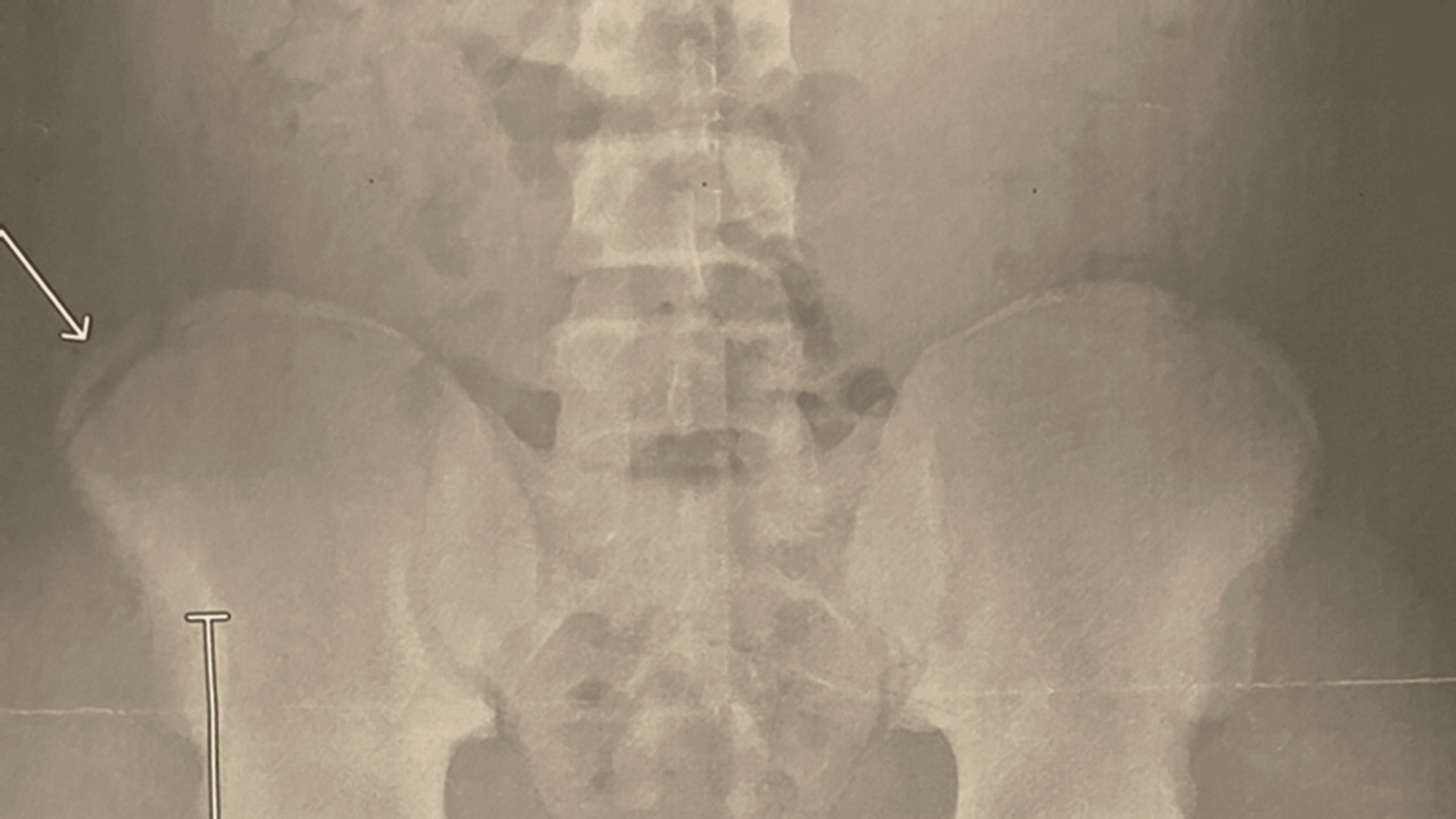
Radiograph of the right iliac crest The arrow denotes the apophyseal injury.

A 16-year-old high school football player presented after 4 days of constant anterior right hip pain that started after feeling a pop while running at full speed during a game. At the time of injury, he was unable to ambulate independently due to pain. His ability to ambulate during the following four days was prevented by pain. Examination demonstrated a left hip shift, anterior right innominate, severe pain on palpation of the right iliac crest, and a positive Ely test. Active range of motion of the right hip was substantially limited secondary to pain for flexion, abduction, extension, and left side bending. Left hip active range of motion was full and nonpainful. Radiography identified a widened cleft in the lateral portion of the apophysis of the right iliac crest (Figure 1). 

Our patient was diagnosed with iliac apophysitis and had a full return to sport after eight weeks of conservative treatment. Follow-up radiography demonstrated the apophysis reuniting with adequate healing. Conservative care consisted of relative rest, activity modification, and physical therapy. Physical therapy was specific for returning to sports for an American Football player.

## Discussion

**Figure 2:**
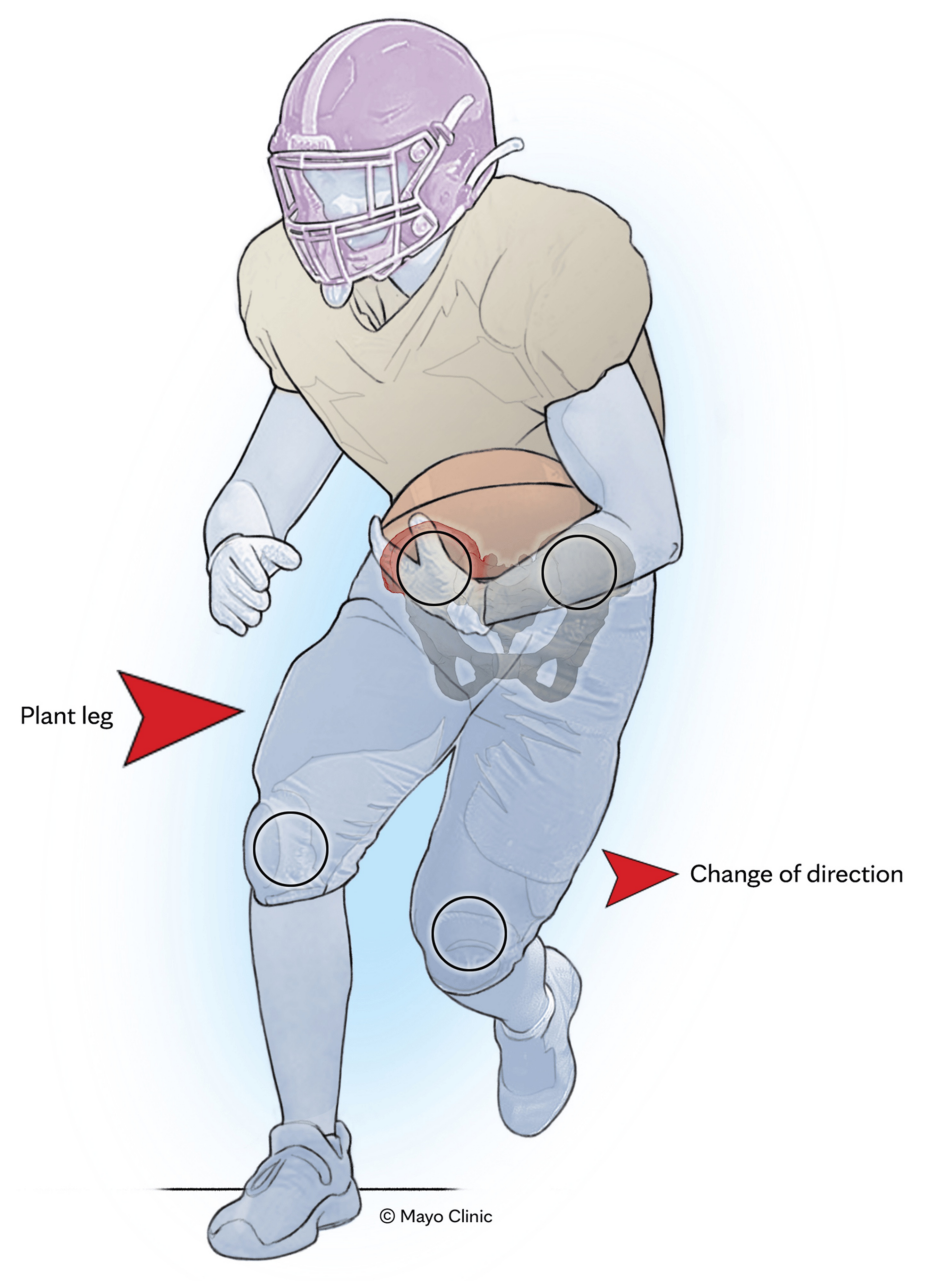
Direction change while running at full speed leading to an overload of eccentric forces at the apophysis, which creates the traction apophysitis injury Used with permission from the Mayo Foundation for Medical Education and Research.

Apophyseal avulsion fractures are unique injuries to secondary ossification centers in children and adolescents. The apophyses are weak points in the muscle-tendon-bone complex and are susceptible to injury from sudden eccentric contractions of attached muscle (Figure 2).

Diagnosis and treatment of these injuries require in-depth knowledge of local anatomy and the mechanism of injury.

**Figure 3:**
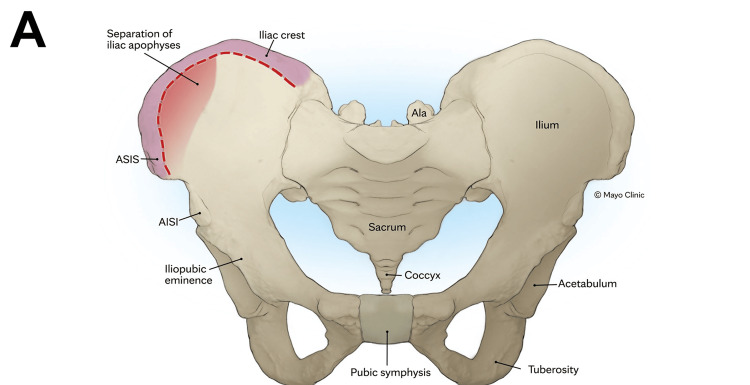
Diagram of the hip and pelvis Diagram showing the separation of the iliac apophyses (red area) seen in traction injuries. AISI: anterior inferior sacroiliac; ASIS: anterior superior iliac spine Used with permission from the Mayo Foundation for Medical Education and Research.

The mean age of apophyseal avulsion fractures of the pelvis is 14.5 years; the most commonly injured apophyses are the anterior inferior iliac spine, ischial tuberosity, anterior superior iliac spine, and iliac crest (Figure 3) [5].

These fractures are noncontact injuries that occur during sudden trunk rotation while an athlete is sprinting [11]. Iliac crest avulsions are typically caused by forceful contraction of the abdominal oblique [8]. These injuries account for 1% to 2% of apophyseal fractures of the pelvis and have been reported in gymnastics, wrestling, and football [10].

Diagnosis of iliac apophysitis

**Figure 4:**
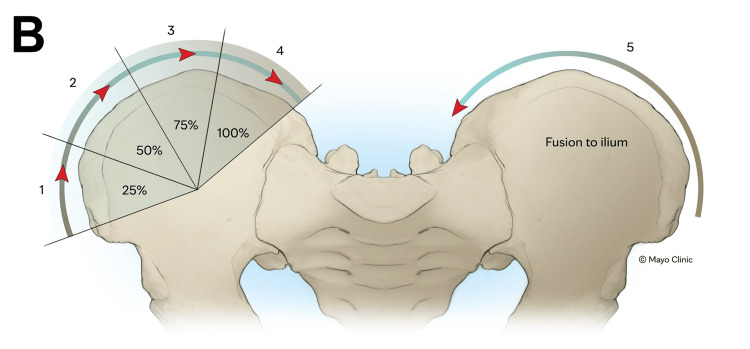
Diagram showing Risser Classification Grade 1, Ossification of the lateral 25% of the iliac apophysis. Grade 2, Ossification of the lateral 50% of the iliac apophysis. Grade 3, Ossification of the lateral 75% of the iliac apophysis. Grade 4, Ossification of the entire (100%) apophysis. Grade 5, fusion of the ossified epiphysis to the iliac wing. Used with permission from the Mayo Foundation for Medical Education and Research.

The diagnosis of iliac apophysitis is based on effective medical history, physical examination, and anterior-to-posterior radiography of the hip [3]. Radiography is used to determine the alignment and separation of bony elements. The skeletal maturity of the patient can be classified using the Risser classification (Figure 4).

This combination of clinical assessment and radiography is usually adequate to correctly make the diagnosis. When radiography is limited due to tissue overlap, magnetic resonance imaging, computed tomography, or ultrasound can be used [3]. Computed tomography can confirm the presence of a displaced ossified apophysis and show callus formation or heterotopic ossification from chronic avulsion injuries [5]. Magnetic resonance imaging can reveal soft tissue injuries, such as musculotendinous strains, edema, hematoma, and avulsion injuries, and retraction of tendons, which can aid in identifying patients who may benefit from surgical intervention [12]. Ultrasound can be used to identify avulsion injuries in the acute care setting but is limited by the user’s ability.

Treatment

Treatment of avulsion injuries to the iliac crest is mostly conservative, consisting of activity modification, pain modulation, and physical therapy. Initially, the patient is advised to rest and use nonsteroidal anti-inflammatory medications and cryotherapy to control pain [13]. Once pain and inflammation are controlled, gentle activity is reintroduced beginning with open-chain exercises to strengthen the lower extremity, core stability training, and gentle range-of-motion exercises [11]. The patient gradually progresses to closed-chain exercises to simulate functional activities and address deficiencies in the patient’s kinetic chain. Finally, sport-specific exercises are introduced [14].

Surgical repair with open reduction and screw fixation is rarely used, approximately in 1% of cases, as outcomes are comparable to conservative therapy [1]. Candidates for surgical repair of an iliac crest apophyseal injury have a gap of more than 15 mm [14]. While the initial recovery period can be shorter with an earlier return to sport, mid- and long-term outcomes generally do not differ from conservative treatment [14].

The overall prognosis for this injury is good. The typical recovery period is 3 to 12 weeks, depending on bony spacing and proper progression through a graded therapy program. The physical therapy protocol focused initially on pain reduction and gentle range-of-motion activities, then it progressed to progressive strengthening of the hip musculature, and finally to sports-specific exercises for American Football. The clinical information that impacted the progress for return to sport activity was no tenderness to palpation at the iliac crest, full and painless range of motion, equal strength compared to the unaffected side, and pain-free sprinting, jumping, and cutting. Early return to sporting activities can slow healing and lead to recurrent apophyseal injuries [14].

## Conclusions

Apophyses avulsions of the hip and pelvis are rare injuries that occur in adolescent athletes. Diagnosis requires in-depth knowledge of the mechanism of injury through physical examination and radiography, and treatment should be tailored to the athlete with education on gradual return to sport to prevent recurrent injuries or delayed healing.
